# Seasonal Spatial Distribution Patterns of the Sand Crab *Ovalipes punctatus* (De Haan 1833) in the Southern Yellow and East China Seas and Predictions from Various Climate Scenarios

**DOI:** 10.3390/biology14080947

**Published:** 2025-07-28

**Authors:** Min Xu, Jianzhong Ling, Haisu Zheng, Xiaojing Song, Zunlei Liu, Huiyu Li

**Affiliations:** 1 Key Laboratory of Fisheries Remote Sensing Ministry of Agriculture and Rural Affairs, East China Sea Fisheries Research Institute, Chinese Academy of Fishery Sciences, Shanghai 200090, China; xuminwzy@aliyun.com (M.X.); lingjz@ecsf.ac.cn (J.L.); songxiaojing@ecsf.ac.cn (X.S.); liuzl@ecsf.ac.cn (Z.L.); 2 Shanghai Aquatic Wildlife Conservation and Research Center, Shanghai 200080, China; zhenghaisu322@hotmail.com

**Keywords:** stock assessment, aquatic animals, conservation, exploitation, crab species, total allowable catch, fishery policy, overfish

## Abstract

In the past two decades, little information has been updated to understand the resource status of the crab species *Ovalipes punctatus* in the East China Sea Region. In this study, we conducted surveys in 2018 and 2019 to identify the seasonal spatial distribution patterns of the economically important sand crab *Ovalipes punctatus* (De Haan 1833) in the southern Yellow and East China Seas. In the study area, the largest biomass of crabs was observed in the fishing grounds of Dasha and the Yangtze River mouth, and the second largest biomass was detected in the Jiangwai-Zhouwai area. Seasonally, the total biomass order in these areas was summer > autumn & winter > spring, and the mean average individual weight order was spring & summer > winter > autumn. These findings provided maps of the seasonal spatial distribution pattern of the species across seasons, which were then used in climate-change scenario models. Model predictions suggested that *O. punctatus* might migrate northward and offshore under climate warming conditions, and that the climate scenario SSP585-2100 might be the most negative case, respectively, for the habitat area of gain% minus loss%. These data can be used to develop robust and systematic regional fisheries resource management policies that consider adaptation measures to address the impact of environmental and climate change along China’s coasts and other areas in the world.

## Simple Summary

Spatial distribution models that consider biotic and abiotic factors are necessary to understand the distribution pattern variations and life history traits of crabs. The sand crab *Ovalipes punctatus* (De Haan 1833) is an economically important species in China, Korea, and Japan, where its market price and demand are very high. In this study, we first collected *O. punctatus* distribution data during cruises conducted in 2018 and 2019 in the southern Yellow and East China Seas. We then used ensemble models to predict seasonal distribution variations and climate scenarios. The results showed that the crabs were concentrated in the northern East China Sea in spring but migrated to offshore areas in the southern Yellow and East China Seas in summer, where they remained in autumn and winter. These results provide important data for developing future climate adaptive fishery management strategies and devising best practices for the application of species distribution models for fishery management and conservation planning.

## 1. Introduction

**Figure 1:**
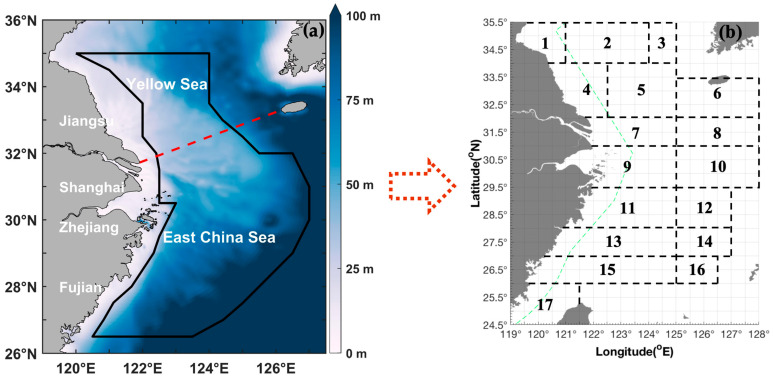
(**a**) Map of the study area (26.50–35.00° N, 120.00–127.00° E), which is denoted by a dark solid line in the East China Sea region and includes the southern Yellow and East China Seas. The color bar denotes a depth range of 0–100 m. The red dashed line indicates the boundary between the Yellow Sea and the East China Sea. (**b**) The black boxes and numbers represent the following fishing grounds: (1) Haizhou Bay, (2) Lianqingshi, (3) Liandong, (4) Lvsi, (5) Dasha, (6) Shawai, (7) Yangtze River mouth, (8) Jiangwai, (9) Zhoushan, (10) Zhouwai, (11) Yushan, (12) Yuwai, (13) Wentai, (14) Wenwai, (15) Mindong, (16) Minwai, and (17) Minzhong.

The sand crab species *Ovalipes punctatus* (De Haan 1833) occurs mainly in the Yellow and East China Seas of China, in coastal areas of Japan, Korea, and Australia, and in the Indian Ocean [1]. This species is a bycatch target of beam shrimp trawling and crab pot fisheries [1]. It has a life span of almost 15 months and prefers to inhabit sandy bottoms in shallow areas (~5–60 m), although it has been found at depths of 95 to 140 m in offshore areas of the Mindong fishing ground in China (Figure 1) [2]. *O. punctatus* can adapt to a range of temperatures and salinities and has strong swimming abilities [3]. On average, the carapace length and width of this crab are 32.15 mm and 55.5 mm at first sexual maturity, with a wet weight of 45.46 g [4]. However, the minimum size at sexual maturity was a carapace of 63 mm and a wet weight of 76 g in the Mindong offshore fishing ground [5].

In the Yellow and East China Seas, *O. punctatus* accounts for about half of the total catch in spring and winter each year [1]. The catch of this species has gradually increased since the 1980s [6]. Increasing market demand has led to overfishing in China, raising concerns about recruitment failure [7]. In addition, Wang et al. (2011) detected a tendency for individual size miniaturization with an accelerated growth rate in this species [8]. However, despite the high consumption and price of *O. punctatus* in China, Korea, and Japan, studies on seasonal spatial distribution variations in China over the past 20 years have not been conducted.

In the East China Sea, the potential fishery resource of *O. punctatus* was estimated to be 84,092.3 tonnes [9]. Recently, marine crustaceans constituted ~19.2% of the total marine fishery catches of all fishing species in China, equating to about 10 million tons [10]. Among them, crab species are economically and ecologically important in coastal ecosystems, and their seasonal spatial distributions are pivotal for fishery management and conservation. Shifts in the range of habitat area and habitat loss caused by climate change may also reduce crab catches and ultimately negatively impact regional fishing activities.

The massive use of fossil fuels increases the atmospheric CO_2_ concentration and leads to global climate warming, which results in increased ocean temperatures and oceanic CO_2_ concentrations [11]. Such changes can affect the distribution of marine fauna and the ecosystems in which they live [12]. Temperature and CO_2_ changes may decrease the appropriate habitat area for all marine biological communities, cause local species extinctions, increase habitats for non-native species, and promote the expansion and establishment of invasive species [13,14,15]. Climate change is also likely to contribute to direct or indirect variations in the biomass, reproduction, and distribution patterns of marine fauna [16].

The surface water temperature along the eastern coast of China is expected to exhibit a warming trend over the next 80 years [17]. By the end of the twenty-first century, the average ocean surface temperature is predicted to increase by 1.8 to 4 °C in China’s seas [18]. In recent decades, the bottom water temperature in the southern Bohai Sea has increased by 0.013 °C annually on average [19]. Thus, understanding the impact of possible climate change scenarios on marine fauna is crucial.

The first goal of this study was to identify the seasonal spatial distribution patterns of *O. punctatus* in the southern Yellow Sea and East China Sea in 2018 and 2019 and their responses to environmental variables (e.g., depth, water temperature, and salinity). The second goal was to develop ensemble spatial distribution models (SDMs) to predict species distribution variations across seasons and climate scenarios (current, SSP1-2.6, SSP2-4.5, SSP3-7.0, and SSP5-8.5) for 2040–2050 (the 2050s) and 2090–2100 (the 2090s). The findings of this study provide baseline data for the development of fisheries policies to conserve and manage this species and prevent resource depletion (e.g., taking actions to limit total allowable catches (TACs) and closed fishing seasons).

## 2. Materials and Methods

### 2.1. Sampling and Survey Procedures

We performed fishery-independent bottom-trawling surveys from 2018 to 2019 in the southern Yellow and East China Seas (Figure 1). The coastal currents in China carry low-salinity water southward along the coastlines. When the northeast monsoon prevails in winter, the China coastal currents are driven through the Taiwan Strait and into the South China Sea. During the southwest monsoon, which prevails in summer, the China coastal current direction in the southern East China Sea is reversed, flowing north into the East China Sea.

The surveys were conducted using a trawl net (Zhejiang, China) with a cod end mesh size of 20 mm and a height of 10–15 m that was towed by the fisheries research vessels Zhongkeyu 211 and 212) in autumn (2–11 November, 2018), winter (4–27 January, 2019), spring (22 April–10 May 2019), and summer (13 August–27 September 2019). The bottom trawls were conducted in a grid characterized by a latitude and longitude spacing of 30 min × 30 min. Each bottom tow was assigned to a specific grid cell according to the survey location. The average trawl speed was 3 knots, and all tows were conducted for approximately 1 h at each station using a trawl net with a headline of 72.24 m and a groundline of 82.44 m. In total, 519 valid tows were included in this study (127 stations in autumn, 111 stations in winter, 141 stations in spring, and 140 stations in summer).

Samples from each survey station were transported to an indoor laboratory for species identification. The catch per unit effort (CPUE) of each survey was determined in terms of seasonal abundance (CPUE_n_; ind∙h^−1^), and the seasonal catch per unit effort by weight (CPUE_w_; g∙h^−1^) was determined by measuring individuals to the nearest 0.10 g of wet weight. The average individual weight (AIW) was defined as CPUE_w_ divided by CPUE_n_ at each station.

In autumn, the total CPUE_w_ was 11,877.19 g·h^−1^, and the total CPUE_n_ was 503.3 ind·h^−1^. In winter, the values were 19,398.8 g·h^−1^ and 571.77 ind·h^−1^. In spring, they were 8508.74 g·h^−1^ and 200.33 ind·h^−1,^ and in summer, they were 57,428.81 g·h^−1^ and 2195.91 ind·h^−1^.

The equations used to calculate the growth of *O. punctatus* were as follows: Weight (W)_♀_ = 0.0004 × Length (*L*)^2.8121^ and W_♂_ = 0.0006 × (*L*)^2.7358^ in the Zhoushan fishing ground [8]; W = 1.6576 × 10^−4^ × (*L*)^3.0756^ in the Mindong fishing ground [2]; W_♀_ = 1.009 × 10^−4^ × (*L*)^3.1728^ and W_♂_ = 1.7041 × 10^−4^ × (*L*)^3.0632^ in the East China Sea [20]; and W_♀_ = 8.77 × 10^−4^ × (*L*)^2.673^ and W_♂_ = 3.37 × 10^−4^ × (*L*)^2.9073^ in the offshore areas of the Mindong fishing ground [5].

Environmental factors were measured at each station using an SBE-19 profiler (Sea-Bird Scientific, Bellevue, WA, USA), which was calibrated before each survey. Sea surface salinity (SSS) and sea surface temperature (SST) were measured at 3 m below the surface, whereas sea bottom salinity (SBS) and sea bottom temperature (SBT) were measured 2 m above the sea bottom at depths < 50 m and 2–4 m above the bottom at depths > 50 m.

### 2.2. Ensemble Model

SDMs are commonly used in biodiversity and ecological studies to predict the potential distribution of species [21]. SDMs have been applied to estimate the distribution variations of crabs inhabiting different habitats (e.g., estuaries, intertidal zones, and mangrove areas), monitor crab invasions, forecast the locations and center areas of fishing grounds, reflect modifications of typical habitats, identify habitat suitability, and standardize CPUE [22]. SDMs can also be used to assess the possible effects of climate change on the distribution of marine species and provide good predictions of key habitat variables [22].

In this study, we used 10 algorithms to forecast the habitat distribution of *O. punctatus* using our survey data: extreme gradient boosting training (XGBOOST), surface range envelope (SRE), random forest (RF), multiple adaptive regression splines (MARS), generalized linear models (GLMs), generalized boosting models (GBMs), generalized additive models (GAMs), flexible discriminant analysis (FDA), classification tree analysis (CTA), and artificial neural networks (ANNs).

We used the “biomod2” package on the ensemble SDM platform (4.3-4) (https://biomodhub.github.io/biomod2/, accessed on 22 June 2025). We used the mean survey data collected over four months (November 2019 and January, May, and August 2019) to produce the annual model, and we used different seasonal data to produce the seasonal models. Future climate data were obtained from the Coupled Model Intercomparison Project Phase 6 (CMIP6), and environmental data (SSS, SBS, SST, and SBT) were obtained from the website Bio-ORACLE (https://bio-oracle.org/index.php, accessed on 22 June 2025). The four Shared Socioeconomic Pathways (SSPs) scenarios (SSP126, SSP245, SSP370, and SSP585) for 2040–2050 (the 2050s) and 2090–2100 (the 2090s) were used in this study [23].

## 3. Results and Discussion

### 3.1. Seasonal Spatial Distribution Characteristics and Patterns

**Figure 2:**
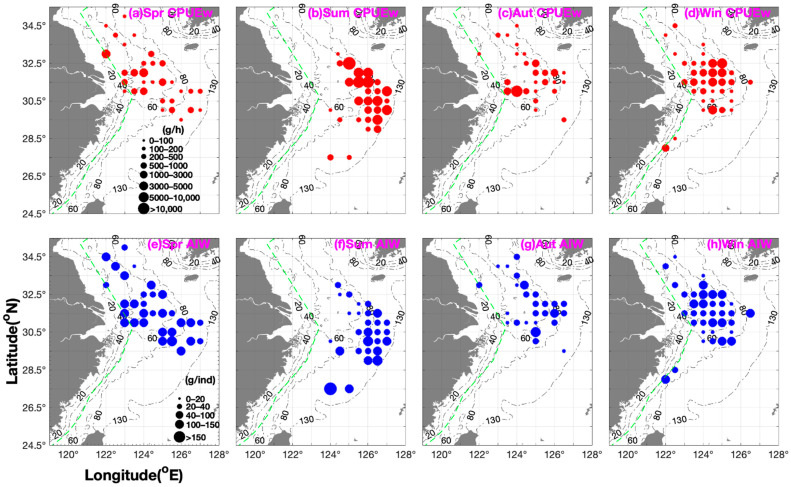
Seasonal distribution patterns of *Ovalipes punctatus* catch per unit effort by weight (CPUE_w_; g·h^−1^) are shown in red (grouped into 0–100, 100–200, 200–500, 500–1000, 1000–3000, 3000–5000, 5000–10,000, and >10,000 g·h^−1^), and AIW (g·ind^−1^) data are shown in blue (grouped into 0–20, 20–40, 40–100, 100–150, and >150 g·ind^−1^). (**a**–**d**) CPUE_w_ in (**a**) spring, (**b**) summer, (**c**) autumn, (**d**) winter; (**e**–**h**) AIW in (**e**) spring, (**f**) summer, (**g**) autumn, and (**h**) winter.

Figure 2 shows the seasonal spatial distribution patterns of *O. punctatus* in the study area. The biomass and number of *O. punctatus* in different seasons in different fishing grounds were in the following order: Dasha > Yangtze River mouth & Jiangwai > Zhouwai > Lianqingshi in spring; Shawai > Jiangwai > Zhouwai > Wenwai-Minwai in summer; Yangtze River mouth > Shawai > Zhouwai > Lianqingshi in autumn; and Dasha > Yangtze River mouth > Yushan > Zhoushan > Lianqingshi in winter (Figure 1 and Figure 2 & Table 1). The seasonal longitudinal order of the biomass was as follows: 124° E–124.5° E > 122° E–123.5° E > 125° E–125.5° E > 126° E–127° E in spring; 125° E–125.5° E > 127° E > 126° E–126.5° E > 124° E–124.5° E in summer; 124° E–124.5° E > 125° E–126.5° E > 122° E–123.5° E in autumn; and 124° E–124.5° E > 125° E–126.5° E > 123° E–123.5° E > 122° E–122.5° E in winter (Figure 1 and Figure 2), indicating the major biomass in the offshore area of 124° E–124.5° E. Table 2 shows the mean seasonal data for CPUE_w_, CPUE_n_, and AIW from autumn 2018 to summer 2019. Seasonally, the order of biomass value was summer > autumn and winter > spring (Table 2). The upper limit value was highest in summer, followed by autumn, then winter, and then spring, indicating a gradual decrease in biomass from summer to spring of the following year (Table 2). The number of *O. punctatus* also showed the order summer > autumn & winter > spring (Table 2).

In spring, the greatest biomass occurred at 20–40 m when CPUE_w_ was >1000 g·h^−1^. In summer, the values were 40–100 m when CPUE_w_ was >1000 g·h^−1^, in autumn they were 40–70 m when CPUE_w_ was >200 g·h^−1^, and in winter they were 40–55 m when CPUE_w_ was >1000 g·h^−1^. The groups of crabs with AIW > 50 g·ind^−1^ were found at 40–100 m and those with AIW < 10 g·ind^−1^ were found at 70 m in spring; 80–120 m and 60 m in summer; 60–70 m and 66 m in autumn; and 45–70 m and ~60 m in winter (Figure 2).

Yu et al. (2005) reported that the seasonal order of *O. punctatus* biomass from 1998 to 2000 was spring > summer > autumn > winter and that for number, the order was spring > autumn > summer > winter in the study area [1]. They concluded that the spawning period occurs from February to June, as they found more and more juveniles from July to August, with a very slow growth speed from October to the following January [20]. The female sexual gonad is visible in spring [20].

In China, coastal fishermen mainly target spawning cohorts from March to August [24]. In the Mindong fishing ground, Ye et al. (2004) detected the greatest and least biomasses in summer and autumn, respectively, with peak fishery catches occurring in spring and summer [5]. Yu et al. (2005) recorded the largest biomass in spring from 1998 to 2000 [1]. However, after approximately 30 years of intense overfishing and summer moratoria on marine fishing since 1995, the largest biomass in our study occurred from August to September 2018 after the end of the summer fishing moratorium. The annual mean individual weight was 41 g·ind^−1^ in our study compared to 39 g·ind^−1^ from 1998 to 2000 [24].

Yu et al. (2006) reported that the areas with the largest biomass of *O. punctatus* included 31.5° N–32.5° N, 122.5° E–124° E (fishing ground of the Yangtze River mouth); 30° N–30.5° N, 125° E–126.5° E (~80 m depth in the Zhouwai fishing ground); and 26° N–27° N, 122° E–123° E (~80–120 m depth in the Mindong fishing ground) [24]. They concluded that the northern East China Sea is this crab’s spawning and nursery ground, with the center of the grounds located at < 40 m depth in the southwestern Dasha and northwestern Yangtze River mouth fishing grounds in the range of 31.5° N–33° N, 122.5° E–124° E [24]. They also concluded that the central distribution area is located in the southwestern Dasha fishing ground in spring, with the order as follows: Dasha & Yangtze River mouth > Zhouwai > Mindong [24].

Our study results suggest that the central distribution area is located at 31° N–33° N, 122.5° E–126° E (Figure 2). We also found an intense distribution at a depth of ~ 80 m in the Zhouwai fishing ground in spring, but we did not find major biomass in the Mindong fishing ground (Figure 1 and Figure 2). In summer and autumn, the majority of the biomass occurred in offshore water areas (Figure 2).

The mean individual size of the species in the fishing grounds was as follows: Zhouwai > Yangtze River mouth & Jiangwai > Dasha > Lianqingshi in spring; Wenwai-Minwai > Zhouwai > Jiangwai > Dasha & Shawai in summer (with the smallest individual found in the southern Yellow Sea and the largest in the East China Sea); Dasha & Shawai > Yangtze River mouth & Jiangwai > Lianqingshi > Zhouwai in autumn; and Yushan > Dasha & Yangtze River mouth > Lianqingshi > Zhoushan in winter (Figure 1 and Figure 2; Table 1). The longitudinal order of the individual size was as follows: 125° E–125.5° E > 126° E–127° E > 122° E–123.5° E > 124° E–124.5° E in spring; 126° E–126.5° E > 124° E–124.5° E > 127° E > 125° E–125.5° E in summer; 124° E–124.5° E > 126° E–126.5° E > 125° E–125.5° E > 122° E–123.5° E in autumn; and 122° E–122.5° E > 125° E–125.5° E > 123° E–123.5° E > 124° E–124.5° E in winter (Figure 1 and Figure 2). The mean AIW showed the order of spring and summer > winter > autumn, and the upper limit value of AIW was in the order of summer > autumn > spring > winter (Table 2). Yu et al. (2006) suggested that most of the larger crabs occur in the shallower water adjacent to the inshore area in spring, and they also found juvenile and parent cohorts in spring, with the size order of summer > winter > spring [24].

### 3.2. Seasonal Variations of Environmental Variables

Table 3 presents the seasonal data for the environmental factors. SST values were similar in spring and winter, but the lower limit of SST in winter was lower than that in spring (Table 3). In summer, *O. punctatus* were found in warmer water areas with SST from 26 °C to 30 °C, and then in autumn, SST decreased (Table 3). SSS was similar in autumn, winter, and spring, but it increased during the transition from autumn to winter, and the cohort migrated to offshore areas at this time (Table 3). From winter to spring, SSS decreased, and the cohort gradually migrated to the inshore areas (Table 3).

The lower limit value of SBT was in the order of summer > spring > winter > autumn, and the upper limit value of SBT was in the order of summer > autumn > spring > winter (Table 3). The SBS values were similar, and the distribution range of the cohort was similar across the four seasons (Table 3). The depth values at which *O. punctatus* occurred in spring, autumn, and winter were similar, but in summer, they migrated to shallower water areas (Table 3).

**Figure 3:**
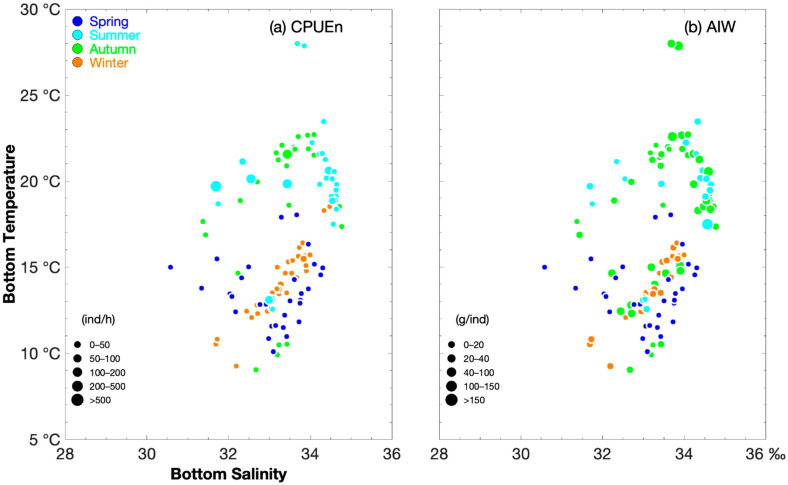
Relationship between salinity (‰) and temperature (°C) for catch per unit effort by number (CPUE_n_) size classified by group (0–50, 50–100, 100–200, 200–500, and >500 ind·h^−1^) and average individual weight (AIW) size classified by group (0–20, 20–40, 40–100, 100–150, >150 g·ind^−1^) of *Ovalipes punctatus*. The data for spring, summer, autumn, and winter are denoted by solid blue, light blue, green, and brown circles, respectively. (**a**) Sea bottom temperature vs. sea bottom salinity for CPUE_n_; (**b**) sea bottom temperature vs. sea bottom salinity for AIW.

Figure 3 shows the relationship between the sea bottom salinity and temperature for CPUE_n_ and AIW. In spring, the greatest number of *O. punctatus* occurred at SBT 13.44 °C–14.38 °C and SBS 32.04‰–32.32‰ when CPUE_n_ was >20 ind h^−1^ (Figure 3). In summer, the values were 19.07 °C–20.61 °C and 31.69‰–34.6‰ when CPUE_n_ was >100 ind h^−1,^ and in autumn, they were 21.56 °C and 33.44‰ when CPUE_n_ was >100 ind h^−1,^ and in winter, they were 13.23 °C–15.47 °C and 33.01‰–33.84‰ when CPUE_n_ was >50 ind h^−1^ (Figure 3).

The groups of crabs with AIW > 50 g·ind^−1^ were found at SBT 11.81 °C–18.03 °C and SBS 30.58‰–34.25‰ in spring; 17.49 °C–28 °C and 33.68‰–34.64‰ in summer; and 12.43 °C–18.52 °C and 32.44‰–34.48‰ in winter (Figure 3). The groups of smaller-sized individuals (<10 g·ind^−1^) occurred at 18.59 °C and 33.48‰ and at 9.92 °C and 33.19‰ in autumn and at 15.64 °C–15.70 °C and 33.71‰–34‰ in winter (Figure 3).

Yu et al. (2005) reported that *O. punctatus* were mainly concentrated at depths of <40 m in spring, >80 m in summer, <40 m in autumn, and 40 to 60 m in winter [1]. Zoeae were detected at a depth of 38 m [25]. Yu et al. (2005) also found that the equation “number = 15,828.1 − 537.77 × water temperature” explained the distribution in summer, with a negative relationship between the individual number of crabs and water temperature in this season [1]. Our results indicated a preference for lower water temperatures, with the majority of the biomass of *O. punctatus* recorded in winter and spring. Yu et al. (2005) found high biomass at temperatures between 12 °C and 15 °C in spring [1]. It was found that the zoeae of *O. punctatus* cannot tolerate water temperature > 27.5 °C and salinity < 15‰ and that the optimal values for zoeae survival were 15–22 °C and 34‰–35‰ [25].

### 3.3. Habitat Predictions Under Different Climate Projections

**Figure 4:**
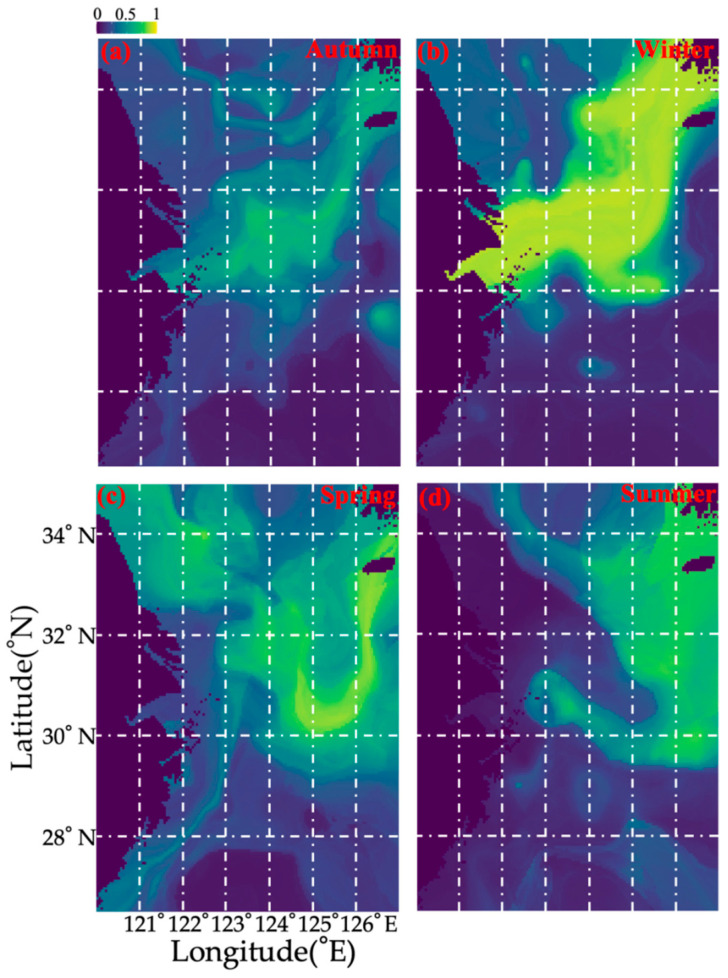
Current seasonal spatial predicted distribution patterns of *Ovalipes punctatus* (**a**–**d**) from spring to winter in the study area based on data collected from 2018–2019.

Figure 4 shows the current seasonal spatial distribution pattern of *O. punctatus*. Our survey data show that in spring, *O. punctatus* were concentrated in the northern East China Sea, including the depth range of 20 to 40 m in Jiangsu coastal water and >40 to 80 m outside the Yangtze River estuary extending to Jeju Island (Figure 4 and Supplementary Figure S1). In summer, the crabs moved to offshore areas with depths < 60 m in the southern Yellow and northern East China Seas (29.5° N to 33° N) (Figure 4 and Supplementary Figure S1). In autumn, they were concentrated in the northern East China Sea, including the Shawai, Jiangwai, and Yangtze River mouth fishing areas (Figure 4). In winter, the crabs remained in the Dasha, Shawai, Jiangwai, and Yangtze River mouth fishing grounds with an enlarged distribution range (Figure 4 and Supplementary Figure S1).

**Figure 5:**
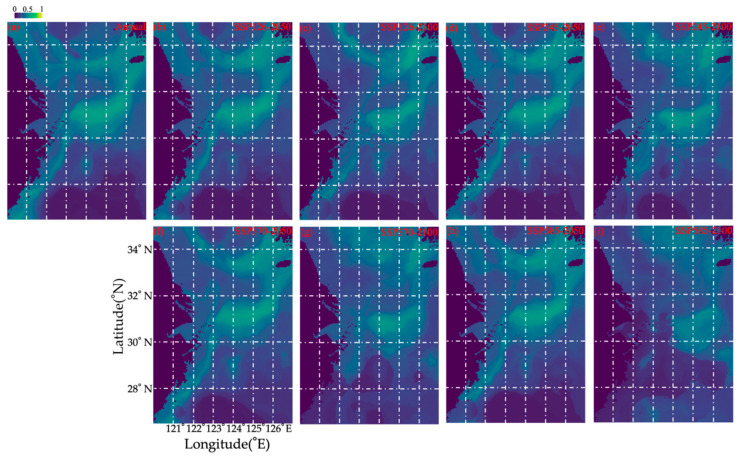
Predicted spatial habitat distribution patterns of *Ovalipes punctatus* in the cases of (**a**) annual mean habitat, (**b**) SSP126 in 2050, (**c**) SSP126 in 2100, (**d**) SSP245 in 2050, (**e**) SSP245 in 2100, (**f**) SSP376 in 2050, (**g**) SSP370 in 2100, (**h**) SSP585 in 2050, and (**i**) SSP585 in 2100.

Currently, *O. punctatus* is concentrated in the southern Yellow and East China Seas, including the fishing grounds of Jiangwai and Shawai and the areas near Jeju Island (Figure 5 and Supplementary Figure S2). A comparison of this case with that of the SSP126-2050 scenario showed that the distribution range in Jiangsu offshore waters would decrease. The distribution ranges in the SSP126, SSP245, SSP370, and SSP585 scenarios for 2050 were similar. An obvious northward distribution tendency was detected in the SSP370-2100 and SSP585-2100 scenarios compared with the SSP126-2100 and SSP245-2100 ones. In the SSP370 and SSP585 scenarios, *O. punctatus* were distributed in water > 60 m depth in the southern Yellow Sea; however, in the case of SSP370, they remained in the fishing grounds of the Yangtze River mouth and Jiangwai, but under SSP585, they gradually migrated to the Jiangwai fishing ground, showing a tendency for offshore movement (Figure 5 and Supplementary Figure S2).

The models provided various scenarios for future habitat loss and gain (Table 4). The loss area was in the order of SSP585-2100 > SSP370-2100 > SSP126-2100 & SSP245-2100 > SSP370-2050 & SSP585-2050 > SSP126-2050 & SSP245-2050, indicating a greater loss of area in 2100 than in 2050 (Table 4). Greater loss was predicted for SSP585 and SSP370 than for SSP126 and SSP245 (Table 4). The gain area was in the order of SSP245-2100, SSP370-2100, and SSP585-2100 > other scenarios, indicating a greater predicted gain of area in 2100 (Table 4). The “gain minus loss” analysis showed that the population would gradually move northward and to offshore areas in the order of SSP245-2050 > SSP126-2050 & SSP245-2100 & SSP370-2050 & SSP585-2050 > SSP370-2100 & SSP126-2050 > SSP585-2100 (Table 4).

### 3.4. Implications for Fisheries Management, Underlying Benefits, and Future Studies

Numerous impacts of global warming are expected, including shifts in the distribution of various marine species [26]. The distribution area of Pacific cod (*Gadus microcephalus*) in the Yellow Sea has moved northward by 0.5° N since 1959 [27]. Climate change is likely to affect the life histories and cycles of marine species [28]. Coastal nearshore warming could be more evident than that in deep water, and the warming rate will likely occur more rapidly at higher latitudes than at lower latitudes [29]. Climate change could reduce the suitable habitat areas for *O. punctatus* in the study area and force them to migrate to higher latitudes. Our data showed a trend of habitat reduction from low to high latitudes and from nearshore to deep-sea areas for *O. punctatus*. Changes in distribution patterns may cause community restructuring and ecosystem biodiversity loss, which in turn could have serious impacts on marine fisheries [30]. A series of refuges for the studied species under continuous climate warming needs to be planned in the current management actions, which is a very important factor in regulating crab recruitment in natural populations [31].

Gao’s findings support the conclusion of a single-stock management regime for the species within the Yellow and East China Seas [7], but Yu et al. (2010) argued that the species in this area should be separated into at least two management units [32]. In 2000, China’s central government revised the Chinese Fisheries Law and clearly introduced the idea of a quota fishing system. This system is an active management system that sets a maximum allowable catch for specific fishery resources within a specific time and area. Subsequently, regulations were formulated to gradually implement a quota fishing system to strictly manage and reduce the number of fishing vessels in coastal waters. Future works are needed to identify the accurate management units for the species in the study area. Currently, the system is hindered by the absence of real-time and long-term monitoring to obtain scientific data and the inability to ensure the actual catch or landed quantity within the set TAC range. In response, the government has strictly implemented actions such as summer fishing moratoria and minimum mesh size to protect the juvenile and parent cohorts of marine economic animals.

The results of this study provide a scientific basis for the formulation of an improved fishery management system that includes managing the time, effort, and location of fishing, protecting important habitats, monitoring environmental variations of habitats that may be lost under climate change, and utilizing cohorts in stable habitats in moderation. Our findings also provide important insights into the threat of climate change to suitable habitats for *O. punctatus*.

The geographic distributions of other species in the study area are also likely to change in response to climate change, thereby affecting the distribution and catch of the target species of existing fisheries. Moreover, climate fluctuations during the spawning season can negatively impact recruitment. In the future, an adaptive science-based fishery management strategy that incorporates multiple species should be developed to gather information, predict outcomes, and monitor and evaluate results. A continuous review of what works and what fails should lead to more economically, ecologically, and socially optimal outcomes under changing environmental and climate conditions.

Finally, our study has some limitations. Specifically, the methods used in this study are subject to the risk of overfitting when predicting future distributions of species under different climate scenarios [33].

## 4. Conclusions

Compared to the resource status of *O. punctatus* in 2000, the highest recorded biomass shifted from winter and spring to the end of the summer fishing moratorium (August to September) in our 2018-2019 study. This finding indicates the success and shortcomings of such legal regulations. In addition, we argued that the major biomass of the species concentrated on the Dasha fishing ground for spawning in spring, and migrated south to the Shawai and Jiangwai fishing grounds for feeding and nursery in summer, and concentrated on the Yangtze River mouth fishing ground for food and growth in autumn, and gradually migrated from Zhoushan to Dasha fishing grounds for the preparations of next generation release. The findings of our study have implications for fisheries management and the establishment of new regulations in China and lay the groundwork for sustainable *O. punctatus* fisheries.

## Figures and Tables

**Table 1:** Mean and total values of catch per unit effort by weight (CPUE_w_; g·h^−1^), percentage of CPUE_w_, catch per unit effort by number (CPUE_n_; ind·h^−1^), percentage of CPUE_n_, average individual weight (AIW; g·ind^−1^), and percentage of AIW in different fishing grounds according to season.

	Mean Value					Total Value					
	B	B%	N	N%	AIW	B	B%	N	N%	AIW	AIW%
	Spring										
(2)	70.3	5.9%	1.5	5.5%	38.2	281.4	3.3%	6	3%	152.7	9.6%
(5)	509	42.5%	14	51.2%	41.4	4581.3	53.8%	126.2	63%	372.8	23.3%
(7)	233.5	19.5%	4.2	15.3%	53.2	1401.3	16.5%	25.2	12.6%	319.4	20%
(8)	225.1	18.8%	5.4	19.6%	42.5	1125.5	13.2%	26.9	13.4%	212.3	13.3%
(10)	159.9	13.3%	2.3	8.4%	77.2	1119.3	13.2%	16	8%	540.1	33.8%
	Summer										
(5)	262.1	2.3%	15.2	3.1%	18.9	524.2	0.9%	30.5	1.4%	37.7	2.7%
(6)	7155.8	61.8%	320.8	65.9%	21.8	21,467.4	37.4%	962.4	43.8%	65.5	4.7%
(8)	2434.8	21%	117.8	24.2%	31.4	17,043.6	29.7%	824.8	37.6%	219.5	15.7%
(10)	1440	12.4%	30.8	6.3%	45	17,279.8	30.1%	369.7	16.8%	539.8	38.7%
(14)&(16)	278	2.4%	2.1	0.4%	133.3	1113.8	1.9%	8.5	0.4%	533.4	38.2%
	Autumn										
(2)	62.3	2.2%	3.8	3.1%	16.3	187	1.6%	11.5	2.3%	48.8	7.8%
(5)	60.1	2.1%	2.1	1.7%	32.8	300.3	2.5%	10.5	2.1%	164.2	26.3%
(6)	443.6	15.6%	16.6	13.6%	26	2218	18.7%	82.8	16.4%	130.2	20.9%
(7)	2009.2	70.7%	87.5	71.7%	18.5	8036.8	67.7%	349.8	69.5%	74.2	11.9%
(8)	113.5	4%	4.1	3.4%	29.2	680.8	5.7%	24.9	4.9%	175.3	28.1%
(10)	151.5	5.3%	7.9	6.5%	15.4	454.4	3.8%	23.8	4.7%	30.8	4.9%
	Winter										
(2)	92.8	4.6%	5	8.5%	25.4	185.6	1%	10	1.7%	50.8	4.1%
(5)	820	40.5%	21.2	36.1%	36.8	10,659.8	55%	276	48.3%	478.5	38.2%
(7)	478.4	23.6%	15.6	26.5%	36.2	5740.3	29.6%	187	32.7%	434.5	34.7%
(9)	309.1	15.3%	13	22.1%	23	2163.7	11.2%	90.8	15.9%	161.2	12.9%
(11)	324.7	16%	4	6.8%	63.5	649.4	3.3%	8	1.4%	127.1	10.1%

N.B.: fishing grounds of (2) Lianqingshi, (5) Dasha, (6) Shawai, (7) Yangtze River mouth, (8) Jiangwai, (9) Zhoushan, (10) Zhouwai, (11) Yushan, (14) Wenwai, and (16) Minwai (see Figure 1).

**Table 2:** Seasonal data for catch per unit effort by weight (CPUE_w_; g·h^−1^), number (CPUE_n_; ind·h^−1^), and average individual weight (AIW; g·ind^−1^) from autumn 2018 to summer 2019.

Factor	Spring	Summer	Autumn	Winter
Mean CPUE_w_ at collection stations (g·h^−1^)	274.48	2051.03	456.82	538.86
Value range of CPUE_w_ (g·h^−1^)	11.84–1286.24	18.9–13120	10.95–6896.94	16.06–3450.67
Mean CPUE_n_ at collection stations (ind·h^−1^)	6.46	78.43	19.36	15.88
Value range of CPUE_n_ (ind·h^−1^)	1–44.75	1.09–568	1–282.35	0.97–84
Mean AIW (g·ind^−1^)	51.53	49.85	28.66	34.78
Value range of AIW (g·ind^−1^)	8.97–104.33	3.5–203.17	7.3–121.62	6.7–87.06

Abbreviations: CPUE_w_, catch per unit effort by weight; CPUE_n_, catch per unit effort by number; AIW, average individual weight.

**Table 3:** Seasonal data ranges of environmental factors (SST, SSS, SBT, SBS, depth) in the study area from autumn 2018 to summer 2019.

Factor	Spring	Summer	Autumn	Winter
SST (°C)	12.61–18.02	26.15–29.61	16.97–22.69	9.14–18.41
SSS (‰)	30.58–33.95	27.73–33.97	30.49–34.02	31.6–34.34
SBT (°C)	10.1–18.03	12.55–28	9.04–22.7	9.26–18.52
SBS (‰)	30.58–34.31	31.69–34.65	31.37–34.77	31.69–34.48
Depth (m)	20–105	43–120	21–102	19–90

Abbreviations: SST, sea surface temperature; SBT, sea bottom temperature; SSS, sea surface salinity; SBS, sea bottom salinity.

**Table 4:** Percentages of habitat loss, gain, and overall habitat (gain minus loss) for *Ovalipes punctatus* under various climate scenarios (SSP126-2050, SSP126-2100, SSP245-2050, SSP245-2100, SSP370-2050, SSP370-2100, SSP585-2050, and SSP585-2100).

Case.	Loss%	Gain%	Gain% − Loss%
SSP126–2050	−25.76%	7.77%	−18.00%
SSP126–2100	−38.55%	10.95%	−27.61%
SSP245–2050	−22.22%	9.96%	−12.26%
SSP245–2100	−43.92%	24.11%	−19.81%
SSP370–2050	−31.50%	9.41%	−22.10%
SSP370–2100	−60.09%	28.30%	−31.79%
SSP585–2050	−34.24%	9.62%	−24.62%
SSP585–2100	−78.49%	24.72%	−53.77%

## Data Availability

The original contributions presented in this study are included in the article/Supplementary Material. Further inquiries can be directed to the corresponding author.

## Supplementary Materials

The following supporting information can be downloaded at: https://www.mdpi.com/article/10.3390/biology14080947/s1, Figure S1: The predicted habitat suitability of *Ovalipes punctatus* in different seasons; Figure S2: The predicted habitat suitability of *Ovalipes punctatus* in different climate scenarios.
