# Supplementary material for: Seasonal Spatial Distribution Patterns of the Sand Crab Ovalipes punctatus (De Haan 1833) in the Southern Yellow and East China Seas and Predictions from Various Climate Scenarios
Source: Biology (Basel). 2025 Jul 28;14(8):947. doi: 10.3390/biology14080947 (PMC12383468; doi:10.3390/biology14080947)
Supplement: Supplementary file 1 [file biology-14-00947-s001.zip › biology-3769380-supplementary.pdf]

Supplementary file S1

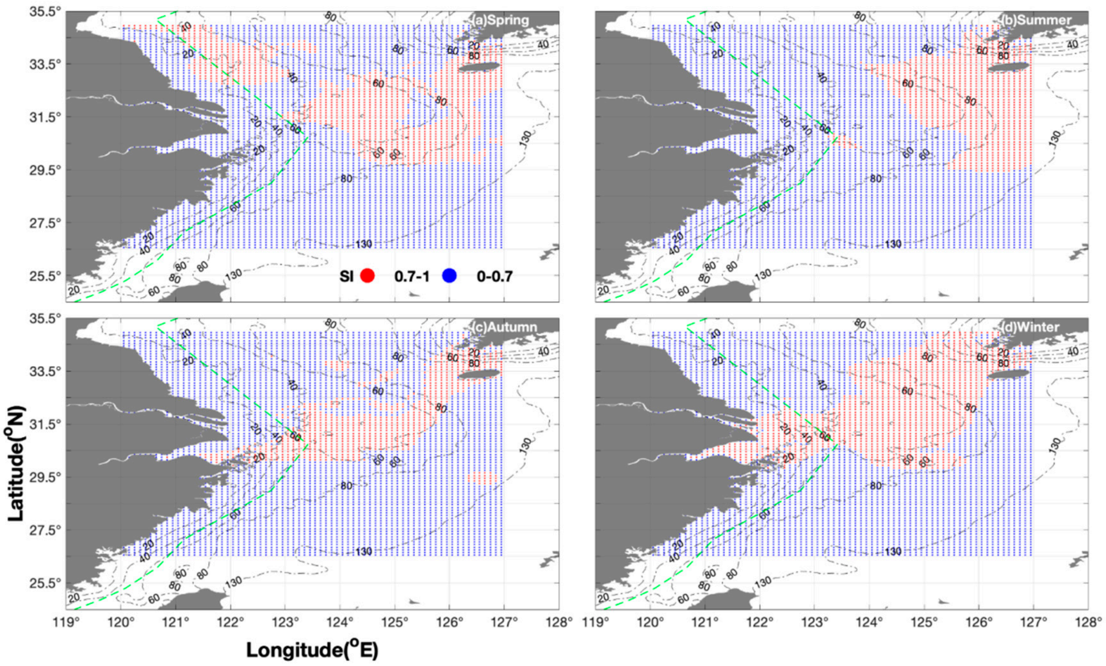

Figure S1. The predicted habitat suitability of *Ovalipes punctatus* in different seasons.

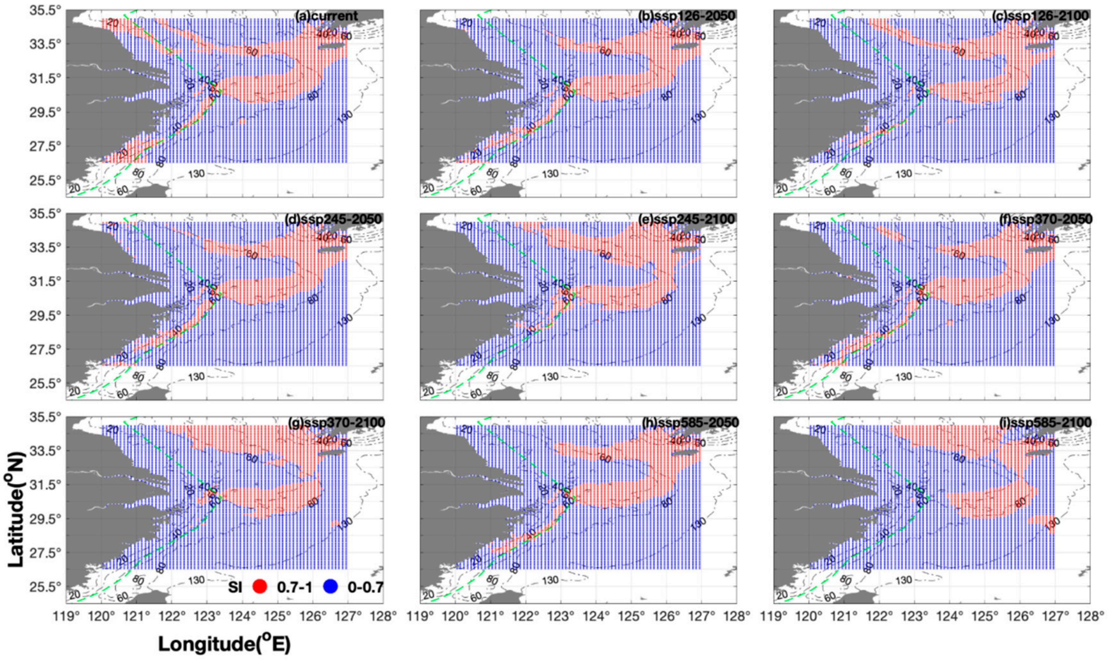

Figure S2. The predicted habitat suitability of *Ovalipes punctatus* in different climate scenarios.
